# A Protein Interaction Map of the Kalimantacin Biosynthesis Assembly Line

**DOI:** 10.3389/fmicb.2016.01726

**Published:** 2016-11-02

**Authors:** Birgit Uytterhoeven, Thomas Lathouwers, Marleen Voet, Chris W. Michiels, Rob Lavigne

**Affiliations:** ^1^Laboratory of Gene Technology, Department of Biosystems, KU LeuvenLeuven, Belgium; ^2^Centre for Food and Microbial Technology, Department of Microbial and Molecular Systems, KU LeuvenLeuven, Belgium

**Keywords:** kalimantacin, yeast two-hybrid, protein interactions, secondary metabolite, antibiotics biosynthesis, batumin

## Abstract

The antimicrobial secondary metabolite kalimantacin (also called batumin) is produced by a hybrid polyketide/non-ribosomal peptide system in *Pseudomonas fluorescens* BCCM_ID9359. In this study, the kalimantacin biosynthesis gene cluster is analyzed by yeast two-hybrid analysis, creating a protein–protein interaction map of the entire assembly line. In total, 28 potential interactions were identified, of which 13 could be confirmed further. These interactions include the dimerization of ketosynthase domains, a link between assembly line modules 9 and 10, and a specific interaction between the *trans*-acting enoyl reductase BatK and the carrier proteins of modules 8 and 10. These interactions reveal fundamental insight into the biosynthesis of secondary metabolites. This study is the first to reveal interactions in a complete biosynthetic pathway. Similar future studies could build a strong basis for engineering strategies in such clusters.

## Introduction

Natural polyketides (PKs) and non-ribosomal peptides (NRPs) are key research objects in view of their potential as therapeutics, including antibacterials, antifungals and immunosuppressant agents. While chemical synthesis approaches to effectively synthesize these complex molecules in the test tube remain challenging and cumbersome, the biological biosynthesis process, using polyketide synthases (PKSs) and non-ribosomal peptide synthetases (NRPSs), has naturally evolved into a very efficient pathway in which multiple PKS and NRPS modules are responsible for the incorporation and/or modification of consecutive building blocks.

In active type I PKS modules, at least three enzymatic domains are present: the acyltransferase (AT) domain selects and recruits an acyl or malonyl coenzyme A building block, the acyl carrier protein (ACP) covalently binds acyl components and the ketosynthase (KS) domain is responsible for the condensation between the growing intermediate and the new building block. However, in so-called *trans*-AT type I PKS clusters, the AT domain is a discrete protein that acts *in trans* to deliver the new building block (Piel, 2010; Helfrich and Piel, 2015). All modules can be further expanded by the presence of a ketoreductase (KR), dehydratase (DH) and/or an enoyl reductase (ER) domain, which change the degree of saturation of the β-carbon of the previous building block, and *cis* methyltransferase (MT) domains can additionally methylate the α-carbon (Smith and Tsai, 2007). Adding to this diversity, *trans*-acting domains, such as oxygenases, reductases, different group transferases, halogenases and cyclases, can further modify the polyketide, both during and after assembly line biosynthesis (Olano et al., 2010). Finally, a thioesterase (TE) domain releases the covalently bound polyketide by hydrolysis, transesterification or by intramolecular cyclization creating linear, macrolacton or macrolactam compounds (Horsman et al., 2015).

Non-ribosomal peptide synthetase modules minimally consist of an adenylation (A) domain, selecting the amino acid building block, a peptidyl carrier protein (PCP) that covalently binds the amino acid precursor and the peptide intermediate, and a condensation (C) domain that connects the new amino acid building block and the growing intermediate. Although most NRPs consist of only 3–15 amino acids, the diversity that can be achieved is astonishing (Mootz et al., 2002). This is due to the capability of the A domains to activate natural and non-natural L- and D-amino acids and the presence of additional modifying domains in *cis* or in *trans*, like epimerization, methylation, cyclization, halogenation or oxidative and glycosylation domains (Samel et al., 2008).

The similar biosynthetic approach in PKSs and NRPSs enables the formation of hybrid clusters and many hybrid PK-NRP compounds have been discovered already (Du et al., 2001). In the past, numerous efforts have been made in finding new PKs, NRPs or hybrid molecules. However, more and more, focus is being shifted toward the engineering of existing gene clusters to create new compounds. The modularity of PKSs and NRPSs seems particularly suited for these engineering approaches and the possibilities of deleting or adding modules or domains, changing the module’s order or combining different gene clusters look to be limitless (Medema et al., 2011). However, these synthetic biology approaches require a relaxed substrate specificity of downstream domains and the different domains or modules need to be able to connect through protein interactions (Giessen and Marahiel, 2012; Wong and Khosla, 2012; Williams, 2013). These requirements appear crucial, since several studies show that the enzyme complexes resulting from manipulated clusters suffer from efficiency problems resulting in low production yields (Gaisser et al., 2003; Menzella et al., 2005, 2007; Kellenberger et al., 2008; Weissman, 2016; Winn et al., 2016). As such, the importance of protein interactions in PKSs and NRPSs systems has been underestimated for many years. Although many researchers have concluded that more insight is needed in protein interactions, only very recently we can see an increase in studies unraveling the modular structure of these systems (Davison et al., 2014; Dutta et al., 2014; Whicher et al., 2014).

**FIGURE 1:**
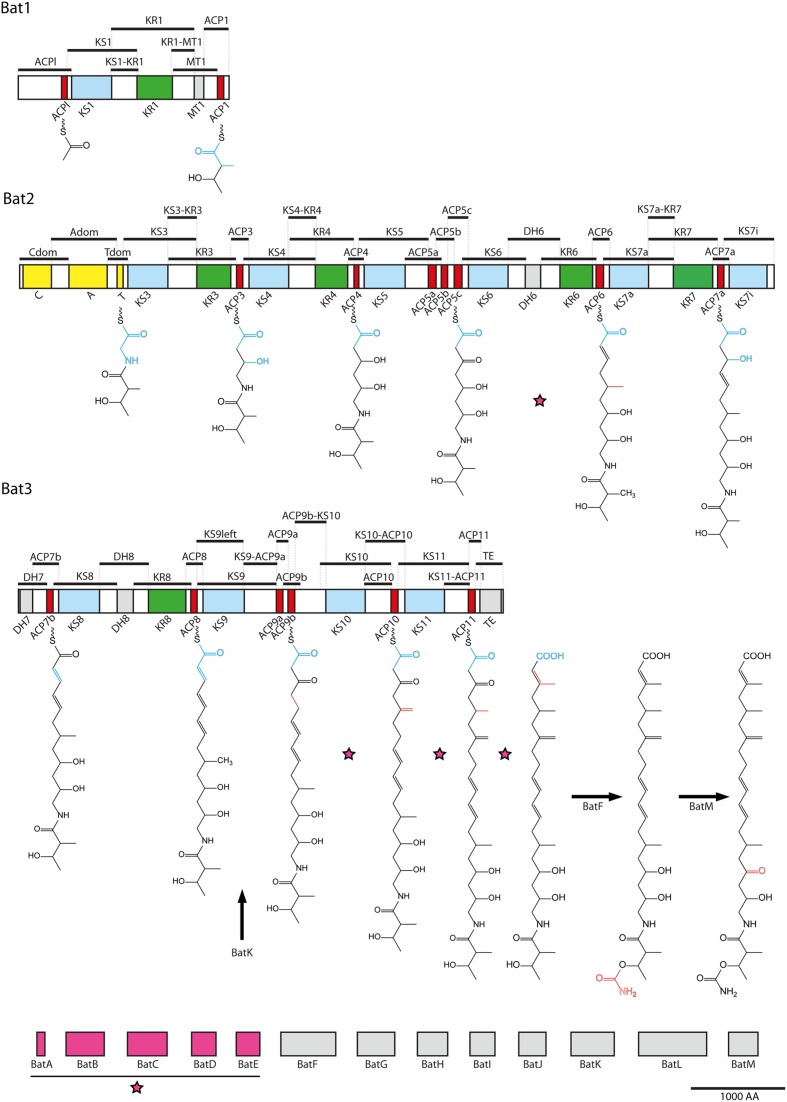
**Overview of fragmentation in the kalimantacin biosynthesis cluster.** Kalimantacin biosynthesis is achieved by PKS and NRPS modules spread across three polypeptides, Bat1–Bat3, and supported by 13 modifying enzymes BatA-M. The NRPS module is colored yellow, while the KS, ACP and KR domains of the PKS modules are marked in blue, red, and green, respectively. Black bars above the proteins represent the 50 regions that form, together with the modifying enzymes, the 63 fragment pool used in Y2H analysis, covering the entire biosynthesis cluster. Modifying enzymes BatA-E assemble into a β-methyl incorporation cassette (pink) that acts four times during assembly of kalimantacin on the sites indicated with a pink star. BatH and BatJ deliver the acetyl- and malonyl-CoA building block to the initiation and elongation modules. BatK, BatF and BatM reduce a double bond, transfer a carbamoyl group and perform oxidation of a hydroxyl group, respectively. New building blocks are illustrated in blue on the molecule’s backbone, while alterations done by modifying enzymes are marked in red.

In this manuscript, a general yeast two-hybrid interaction analysis screen on the kalimantacin assembly line is presented, to gain further insight into the protein interactions in PKSs and NRPSs. Kalimantacin (or batumin) is a hybrid PK/NRP molecule produced by *Pseudomonas fluorescens* BCCM_ID9359 that has strong antistaphylococcal activity (MIC 0.05 μg/ml) and uses FabI as a target (Mattheus et al., 2010a,b). FabI is a trans-2-enoyl-ACP reductase and is essential in the last step of each cycle of fatty acid synthesis (Heath and Rock, 1995). The biosynthesis of kalimantacin is initiated by a *trans*-AT PKS module, followed by an NRPS module incorporating glycine. The rest of the molecule is constructed by ten trans-AT PKS modules, creating the molecule’s backbone and 13 *trans*-acting tailoring domains that deliver building blocks and modify the molecule into the active compound (**Figure 1**) (Mattheus et al., 2010a). The biosynthetic pathway is of special interest for interaction analysis, due to the many features it comprises: both PKS and NRPS modules are present in three different polypeptides, ensuring the presence of both inter and intradomain interactions, two modules consist of multiple ACP domains and different tailoring domains are acting in *trans*.

## Materials and Methods

### Strains and Culture Conditions

*Escherichia coli* Top10 (ThermoFischer scientific, Carlsbad, CA, USA) was used for all cloning purposes and was grown in lysogeny broth (LB) or on LB agar (LB broth with 1.5% w/v agar) at 37°C. *Saccharomyces cerevisiae* AH109 and *S. cerevisiae* Y187 (BD Bioscience) were used in the yeast two-hybrid screen. After transformation, all yeast strains were grown at 30°C on Synthetic Defined (SD) medium (Roucourt et al., 2009), with omission of specific amino acids, dependent on the desired selection, as shown below.

### Cloning Procedures

Open reading frames (ORFs) containing the various domains and inter and intraconnective regions of the kalimantacin assembly line were amplified from the genomic DNA of *P. fluorescens* BCCM_ID9359 using Phusion^®^ High Fidelity DNA polymerase (ThermoFischer scientific). An overview of the primers and the length of the corresponding fragments can be found in Supplementary Table S1. The PCR fragments were inserted in the pCR^TM^8/GW/TOPO^®^ vector (ThermoFischer scientific) by A-overhang ligation. Subsequently, transfer of coding fragments from the TOPO vector to the Gateway^TM^ compatible bait (pGBT9) and prey (pGAD424) vectors (Clontech) was realized using Gateway^®^ LR Clonase^TM^ Enzyme Mix, following the manufacturer’s protocol. All constructs were verified by Sanger sequencing (GATC Biotech).

### Yeast Two-Hybrid Interaction Analysis

*Saccharomyces cerevisiae* AH109 (Mata) and *S. cerevisiae* Y187 (Matα) were transformed with bait and prey vectors, respectively. Transformation of the constructs was performed on 96-well scale, using the protocol of Rajagopala and Uetz (2011). Both yeast strains are auxotrophic for tryptophan, leucine, histidine and adenine. Selection for yeast cells containing the bait vector was performed on SD media lacking Trp, while media without Leu were used for prey selection. Autoactivation of bait constructs was verified by an assay using empty prey vector and prey vector with an unrelated gene from *Pseudomonas aeruginosa* PAO1, *mvaT*. In this assay, the concentration of 3-amino-1,2,4-triazole (3-AT) necessary to reduce leaky expression of reporter genes was optimized at 3 mM.

The actual Gal4p-based yeast two-hybrid screening was performed by an eight-clone pooled, array-based mating screening. First, the bait clones were pooled in groups of eight in such way that every clone was represented in two pools. These pools were mated with the entire prey array, resulting in diploid yeast cells containing one bait and one prey vector. The diploid yeast cells were spotted on SD media lacking histidine and adenine to screen for activation of *HIS3* and *ADE2*. 3-AT and 5-bromo-4-chloro-3-indoyl-α-D-galactopyranoside (X-α-gal) were independently added to the selective media, minimizing leaky expression of *HIS3* and enabling detection of α-galactosidase activity by expression of *MEL1*. The reciprocal screening, prey pool – bait array, was also performed.

Positive interactions were independently confirmed by co-transformation of *S. cerevisiae* AH109 with bait and prey constructs followed by spotting on selective media in twofold dilution series. Finally, the level of the detected protein interactions was quantified using an α-galactosidase assay (Clontech Laboratories, 2009).

## Results and Discussion

### Setup of a High Throughput Interaction Analysis on the Kalimantacin Assembly Line

Yeast two-hybrid screening is a very sensitive and powerful method for detection of protein–protein interactions. Its ability to screen large libraries and even visualize transient interactions makes this technique particularly suited for the analysis of PKS and NRPS systems. However, as an intrinsic limitation of the Y2H approach, expression of bacterial proteins in yeast cells can result in the absence of post-translational modifications present in a natural context, which will impose limitations to the results obtained in the screen. Literature shows that N- and C-terminal fragments of PKS or NRPS domains, often described as linkers and docking regions, are involved in specific interactions connecting modules and domains (Broadhurst et al., 2003; Tang et al., 2007; Buchholz et al., 2009; Cheng et al., 2009). In view of this, delineation of the fragments in this analysis was set up in such way that each domain was flanked by the connector region between two adjacent domains. As such, each flanking region was represented at least two times in the high-throughput screening, as illustrated in **Figure 1**. After amplification, 63 fragments were obtained representing the entire kalimantacin biosynthesis cluster, including tailoring domains BatA-BatM.

### Performing a Pooled Array Screening

First, the 63 fragments were inserted in the pCR^TM^8/GW/TOPO^®^ vector, followed by Gateway transfer to both yeast two-hybrid vectors: bait vector pGBT9 and prey vector pGAD424. Each fragment was tested both as bait and prey protein, thus raising the reliability of the screen. To verify the efficiency of this pooled array approach, a positive control was included in the screen, using interacting proteins Luz24_gp4 (pGBT9) and MvaT (pGAD424) (Wagemans et al., 2015).

The autoactivation assay (Supplementary Table S2) revealed six bait proteins which already activate the three reporter genes (*HIS3*, *ADE2* and *MEL1*) when combined with an empty prey vector or a prey containing the *mvaT* gene. As a consequence, these were excluded from the bait pools. One bait protein, KS1, demonstrated only limited activation of the reporter genes in this control experiment and was retained in the pools.

The remaining bait proteins and pGBT9 positive control were pooled in groups of eight such that every clone was represented in two pools. In total, 16 unique bait pools were created and each pool was separately tested against the entire prey array by mating and testing on selective media. Blue colonies grown on SD-WLHA + X-α-gal plates were considered a positive outcome. By combining the different results, pools interacting with the same prey could be identified. The common bait clone of these pools was determined as the possible interaction partner of that specific prey fragment.

**FIGURE 2:**
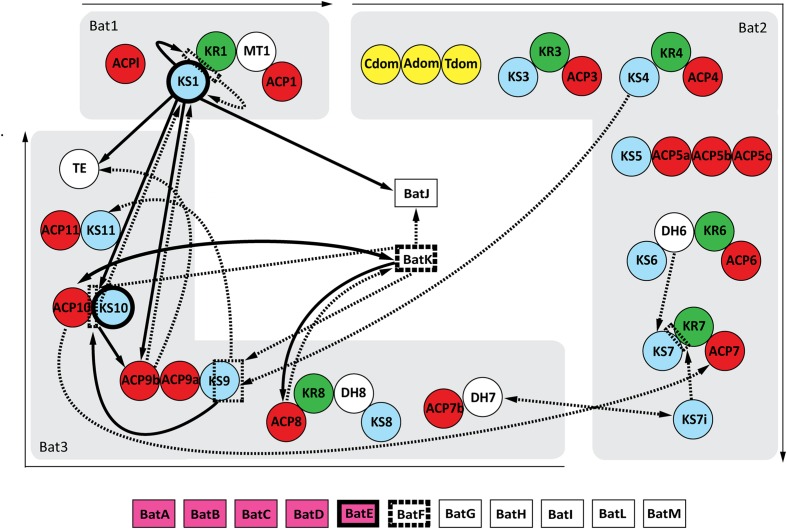
**Interaction map of the kalimantacin assembly line.** Arrows demonstrate the identified protein interactions during Y2H analysis. Single arrows connect the bait protein with its interacting prey partner, whereas double heads indicate interactions found in both the original as reciprocal screen. If the arrow lines are dashed, they represent interactions only found during the initial Y2H screening, whereas protein-proteins interactions shown in full black lines were confirmed during further testing. Domains with a highlighted frame indicate the formation of homodimers.

Twelve possible protein–protein interactions were observed after mating the bait pools with the prey array. The reciprocal screen confirmed 9 of these interactions, but also discovered 16 new possible interacting proteins (**Figure 2**). The positive control was clearly present in both screens, validating the effectiveness of this pooled array screening method.

### Validation of the Discovered Protein–Protein Interactions

Each interaction was verified by co-transformation of *S. cerevisiae* AH109 with the corresponding bait and prey proteins. As a negative control, an unrelated protein Gpa1 was used. In addition, the self-activating bait protein KS1 was tested against MvaT. The screen confirmed 13 interacting pairs, while an additional four bait and prey couples only grew on SD-WLH plates. All validation data and the quantitative interaction data are combined in **Table 1** and are individually discussed and interpreted in the next paragraphs.

**Table 1:** Confirmed interactions observed in the pooled array-based Y2H.

Bait	Prey	SD-WLH + 3-AT	SD-WLHA + X-α-gal	α-Galactosidase units [milliunits/(ml × cell)]
				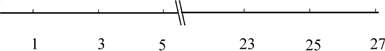					
**ACP – BatK domain interactions**				
ACP8	BatK	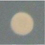	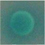	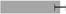					
BatK	ACP8	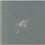	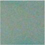	
ACP10	BatK	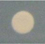	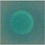	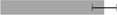					
BatK	ACP10	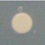	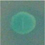	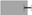					
**Interactions involving the self-activating KS1**				
KS1	BatJ	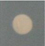	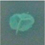	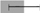					
KS1	KS1	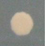	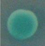	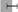					
KS1	KS1-KR1	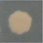	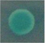	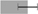					
KS1	ACP9b	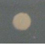	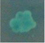	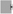					
KS1	KS10-ACP10	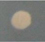	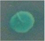	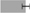					
KS1	TE	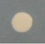	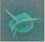	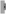					
TE	KS1	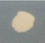	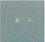	
KS1	MvaT	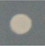	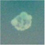	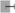					
**Dimer formation**				
BatE	BatE	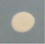	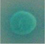	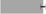					
BatF	BatF	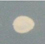	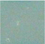	
KS10	KS10	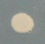	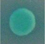	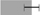					
**Protein interactions between modules 9 and 10**				
KS10-ACP10	ACP9b	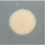	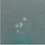	
KS9left	KS10-ACP10	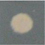	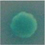	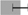					
**Controls**				
Luz24 _gp4	MvaT	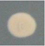	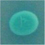	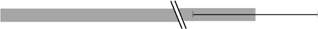					
Gpa1	Gpa1	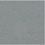	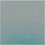	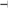					


*All listed protein-protein interactions were confirmed by co-transformation, followed by spotting on selective media. Four interactions only grew on the least selective medium SD-WLH + 3-AT, while the others also displayed a positive outcome in more stringent conditions. These ‘strong’ interactions were further quantified by an α-galactosidase assay, of which the results are graphically displayed as bars.*

### Interactions of the Enoyl Reductase BatK with ACP Domains

The Y2H analysis shows an interaction between the ER BatK and both ACP8 and ACP10. The interaction of BatK with ACP10 can be observed in both directions, whereas ACP8 as bait interacts with the prey construct of BatK, but the reverse direction only results in limited growth on medium lacking histidine (**Table 1**). BatK reduces the double bond created in module 8 by the KR and DH domain (Mattheus et al., 2010a), hence interaction around module 8 is logical. Whether it binds to ACP8, ACP10 or both should be further investigated, but from our analysis it appears that this *trans*-acting enzyme not only recognizes the double bond of the growing intermediate but also attaches to the enzymatic machinery.

### KS1 Potentially Interacts with Several Domains in the Biosynthesis Pathway

KS1 is one of the proteins which shows (limited) autoactivation in this yeast two-hybrid screen. Indeed, the construct in yeast results in a small white colony on the most stringent medium. Hence, results showing big blue colonies, clearly different from the result after the autoactivation test, can be taken into account (**Table 1**; Supplementary Table S2). This strategy resulted in seven potential interaction partners for KS1.

BatJ is predicted to be the *trans*-AT and delivers a malonyl building block to each module (Mattheus et al., 2010a). In this analysis, only an interaction with KS1 is observed. It has been suggested before that *trans*-acting AT domains would dock immediately downstream of the KS domain and it has been observed that the region behind the KS domains in many *trans*-AT systems consists of many residues resulting in unfolded proteins that only adopt a defined fold in the presence of other binding proteins (Cheng et al., 2009; Davison et al., 2014). The interaction of the KS1 fragment with BatJ supports this hypothesis, however, docking of BatJ to this region would be expected to occur in each module. Recently, different studies have shown that *trans*-AT domains can directly transfer their acyl group to the ACP domain, even in the absence of a KS domain or post-KS linker region and suggestions rise that the *trans*-AT domains do not even dock to the mega enzyme cluster (Aron et al., 2007; Musiol et al., 2011; Wong et al., 2011; Ye and Williams, 2014). This could explain the low amount of interactions seen for BatJ.

The interaction observed between KS1-KR1 and KS1 can be explained by the homology of the C-terminal fragment of the KS domain in *trans*-AT PKSs with the so-called KS-AT and post-AT linkers identified in the DEBS KS-AT di-domain structures (Tang et al., 2006, 2007; Davison et al., 2014). These linkers wrap back over the KS domain to stabilize the KS-AT structure. It is possible that the C-terminal fragment here shows comparable behavior and thus interacts with the KS domain. The dimerization of KS1 is discussed further.

The next three protein fragments observed to interact with KS1 are ACP9b, KS10-ACP10 and TE. As they are located at the end of the assembly line, these observed interactions may be false positives. For instance, it is possible for KS1 to interact with the post-KS10 linker region (KS10-ACP10), but since *in vivo*, the post-KS1 linker is covalently attached to the KS1 fragment, the chance of binding this fragment is much higher than the binding to the post-KS10 region. The interaction between KS1 and TE is potentially interesting. Since *in vitro* tests show that the TE-domain does not recognize the ACP domain, but the substrate attached to it, it might now be interesting to specifically look to the interaction between the TE domain and KS domains (Tran et al., 2008, 2010). Still, even if there is interaction between TE domains and KS domains, it is to be expected that this interaction occurs in the last module and not in the first of the assembly line.

The last protein found to interact with KS1 is MvaT. This result is irrelevant for the analysis, since MvaT is an unrelated protein from *Pseudomonas aeruginosa* PAO1. False positive results are inherent to protein interaction techniques, e.g., as a result of artificially combining proteins that can never meet in biological conditions. This result emphasizes the need for focused interaction studies to confirm results obtained in this study by other interaction techniques as discussed in the conclusions and perspectives.

### Dimer Formation in the Kalimantacin Gene Cluster Proteins

In the kalimantacin assembly line BatE, BatF, KS1 and KS10 appear to at least dimerize. BatE is part of the β-methyl incorporation cassette, probably acting with module 5 and modules 9, 10, and 11 (Mattheus et al., 2010a). To our knowledge, no structural data of such cassettes have been described previously. As it is known that a conserved tyrosine residue in helix III of beta-branching ACP domains seems to attract the β-branching cassette and looks to be important for interaction with the cassette, it could be interesting to further explore the structure of such cassettes and to use this information in genetic engineering approaches (Haines et al., 2013).

BatF is the carbamoyl transferase that activates kalimantacin at the end of the biosynthesis pathway by transferring a carbamoyl group to the molecule (Mattheus et al., 2010a). The dimerization observed in this screen could indicate that *O*-carbamoyl transferases are composed as multimers, as has been seen for all *N*-carbamoyl transferase structures identified to date (Shi et al., 2015).

Different structures of PKS modules have been published and it is unclear whether these differences are due to the PKS system analyzed, the method used for structural elucidation or because incomplete PKS modules fold differently than the complete module. However, a common feature in all reported structures is the dimerization of the KS domain (Weissman, 2015). For the kalimantacin cluster, only KS1 and KS10 came back positive for dimerization in this Y2H screen. Although we would expect all KS domains to dimerize, the results are consistent with previous research. It was suggested before that additional dimerization domains in the PKS module help the KS dimerization, since every PKS module has at least one dimerization domain C-terminal of the KS domain (Dutta et al., 2014).

### Interactions Observed between Modules 9 and 10

According to our analysis, modules 9 and 10 interact with each other at two different sites. KS10-ACP10 interacts with the ACP9b fragment, and KS9left with KS10-ACP10. Although modules 9 and 10 are adjacent to each other and interactions are expected, it is difficult to hypothesize on a biological reason for the observed results.

### Some Expected Interactions Remained Unnoticed

Apart from false positive results, it is also possible that interactions that do occur in natural conditions do not appear in the results. These false negative results can, for instance, result from instability of the selected protein fragment, incorrect folding or steric hindrance of the fusion with the activation or DNA binding domain, intrinsically associated with yeast two-hybrid screens (Rajagopala and Uetz, 2009).

The kalimantacin core assembly line consists of three polypeptides. For efficient production of kalimantacin, it would be expected that these proteins interact in a very efficient way. In our results, no interaction between the different proteins is observed. Research on so-called docking domains in the N-terminal and C-terminal region of PKS proteins proved that the dissociation constants of such interactions are very low and thus the proteins interact only transiently (Buchholz et al., 2009; Whicher et al., 2013). Possibly, the interactions are not strong enough to be detected in this pooled assay.

ACP and PCP domains are core proteins in PKS and NPRS clusters, respectively. They carry the new building block and the growing intermediate and are thought to be responsible for the transport of all compounds to the different active sites in the module. Hence, ACP domains are expected to interact with a lot of different proteins. In our analysis, the amount of interactions involving ACP domains was limited. As ACP domains adopt different structures depending on the attachment of the phosphopantetheinyl arm, it can be important that they are in their active *holo*-form for interaction with their partner proteins (Evans et al., 2009). In yeast cells, it is possible that the ACP domains of the kalimantacin cluster do not carry this arm, since not all phosphopantetheinyl transferases show a broad specificity for ACP domains (Lambalot et al., 1996). It might be interesting to repeat the assay described here with a yeast strain heterologously expressing the broad spectrum Sfp phosphopantetheinyl transferase (Quadri et al., 1998).

## Conclusion and Perspectives

The pooled array-based Y2H screening as performed here provides a general first insight in the interactions in the kalimantacin cluster and currently remains the only way to analyze interaction at this scale in a relatively fast manner. Indeed, a total of 63 domains were generated to identify interaction partners in the assembly line, resulting in 3969 tested protein-protein interactions. Based on the analysis, 28 interaction partners were identified, of which 13 pairs could be confirmed independently after co-transformation and an additional four combinations resulted in growth on the least selective medium. In future, the results obtained here can be expanded with other, complementary and more focused interaction analyses, including bacterial two-hybrid and *in vitro* interactomics using pull-down assays.

## Author Contributions

BU and TL contributed equally to this work. BU designed the research and BU, TL, and MV performed the experiments. The article was written by BU and TL. CM and RL actively discussed both design and results with BU and TL. All authors read the work and approved it for publication.

## Conflict of Interest Statement

BU and TL are supported by a FLOF PhD scholarship of the KULeuven. RL and CM are members of the FWO supported Research Community “BaSe-ics.” All the other authors declare that the research was conducted in the absence of any commercial or financial relationships that could be construed as a potential conflict of interest.
