# Supplementary material for: A Protein Interaction Map of the Kalimantacin Biosynthesis Assembly Line
Source: Front Microbiol. 2016 Nov 2;7:1726. doi: 10.3389/fmicb.2016.01726 (PMC5089993; doi:10.3389/fmicb.2016.01726)
Supplement: Supplementary file 2 [file Table_2.DOCX]

Supplementary Material

A protein linkage map of the kalimantacin biosynthesis assembly line

Birgit Uytterhoeven^1+^, Thomas Lathouwers^1+^, Marleen Voet^1^, Chris W. Michiels^2^, Rob Lavigne^1*^

*** Correspondence:** Rob Lavigne: [rob.lavigne@biw.kuleuven.be](mailto:rob.lavigne@biw.kuleuven.be)

+ These authors contributed equally to this work

# Supplementary Figures and Tables

## Supplementary Table 2

**Supplementary Table 2:** Y2H autoactivation assay. Results of testing the bait proteins against an empty prey vector and prey containing *mvaT*, followed by spotting on SD-WLHA + X-α-gal. Both tests resulted in consistent observations.

| **Bait** | SD-WLHA + X-α-gal | **Bait** | SD-WLHA + X-α-gal |
| --- | --- | --- | --- |
| KR3 | 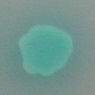 | KS9 | 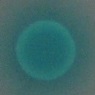 |
| ACP5a | 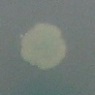 | KS9-ACP9a | 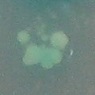 |
| ACP5b | 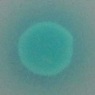 |  |  |
| ACP5c | 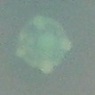 | KS1 | 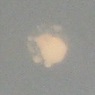 |
